# 2416: A machine learning pipeline to predict acute kidney injury (AKI) in patients without AKI in their most recent hospitalization

**DOI:** 10.1017/cts.2017.75

**Published:** 2018-05-10

**Authors:** Samuel Weisenthal, Samuel J. Weisenthal, Caroline Quill, Jiebo Luo, Henry Kautz, Samir Farooq, Martin Zand

## Abstract

OBJECTIVES/SPECIFIC AIMS: Our objective was to develop and evaluate a machine learning pipeline that uses electronic health record (EHR) data to predict acute kidney injury (AKI) during rehospitalization for patients who did not have an AKI episode in their most recent hospitalization. METHODS/STUDY POPULATION: The protocol under which this study falls was given exempt status by our institutional review board. The fully deidentified data set, containing all adult hospital admissions during a 2-year period, is a combination of administrative, laboratory, and pharmaceutical information. The administrative data set includes International Classification of Diseases, 9th Revision (ICD-9) diagnosis and procedure codes, Current Procedural Terminology, 4th Edition (CPT-4) procedure codes, diagnosis-related grouping (DRG) codes, locations visited in the hospital, discharge disposition, insurance, marital status, gender, age, ethnicity, and total length of stay. The laboratory data set includes bicarbonate, chloride, calcium, anion gap, phosphate, glomerular filtration rate, creatinine, urea nitrogen, albumin, total protein, liver function enzymes, and hemoglobin A1c. The pharmacy data set includes, for each medication, a description, pharmacologic class and subclass, and therapeutic class. Data preprocessing was performed using Python library Pandas (McKinney, 2011). Top-level binary representation (Singh, 2015) was used for diagnosis and procedure codes. Categorical variables were transformed via 1-hot encoding. Previous admissions were collapsed using rules informed by domain expertise (eg, the most recent age or sum of assigned diagnosis codes were retained as elements in the feature vector). We excluded any patient without at least 1 rehospitalization during the time window. We excluded any admission with or without AKI where AKI was also present in the most recent hospitalization. For comparison, we do not exclude such admissions for an identical experiment in which we considered any AKI event as a positive sample (regardless of AKI presence in the most recent hospitalization). We defined an AKI event as an assignment of any of the acute kidney failure (AKF) ICD-9 codes [584.5, AKF with lesion of tubular necrosis, 584.6, AKF with lesion of renal cortical necrosis, 584.7, AKF with lesion of renal medullary (papillary) necrosis, 584.8, AKF with other specified pathological lesion in kidney, or 584.9, AKF, unspecified]. Since diagnosis codes are believed to be specific but not sensitive for AKI (Waikar, 2006), we supplemented them using creatinine for patients who had laboratory values. Diagnosis was made according to the Kidney Disease: Improving Global Outcomes (KDIGO) Practice Guidelines (AKI defined as a 1.5-fold or greater increase in serum creatinine from baseline within 7 d or 0.3 mg/dL or greater increase in serum creatinine within 48 h). We report preliminary model discrimination via area under the receiver operating characteristic curve (AUC) using k-fold cross validation grouped by patient identifier (to ensure that admissions from the same patient would not appear in the training and validation set). It was confirmed that the prevalence of positive samples in the entire data set was maintained in each fold. Python library Sci-kit Learn (Pedregosa, 2011) was used for pipeline development, which consisted of imputation, scaling, and hyper-parameter tuning for penalized (l1 and l2 norm) logistic regression, random forest, and multilayer perceptron classifiers. All experiments were stored in IPython (Pérez, 2007) notebooks for easy viewing and result reproduction. RESULTS/ANTICIPATED RESULTS: There were 107,036 adult patients that accounted for 199,545 admissions during a 2-year window. Per admission, there were at most 54 ICD-9 diagnoses, 38 ICD-9 procedures, 314 CPT-4 procedures, and 25 hospital locations visited. The admissions were 55% female, the average age was 46±standard deviation 20, and average length of stay was 2.5±8.0 days. We excluded 2360 admissions that involved an AKI event that directly followed an admission with an AKI event and 4130 admissions that did not involve an AKI event but directly followed an admission with an AKI event. In total, there were 4561 (5.3%) positive samples (AKI during rehospitalization without AKI in the previous stay) generated by 3699 unique patients and 81,458 negative samples (non-AKI during rehospitalization without AKI in the previous stay) generated by 31,831 unique patients. When using any AKI event as a positive sample (regardless of whether or not AKI was in the most recent stay), the prevalence was 7.3% (6921 positive samples generated by 4395 unique patients and 85,588 negative samples generated by 33,287 unique patients). Best results were achieved with a code precision of 3 digits for which we had a total of 4556 features per patient. Fitted hyper-parameters corresponding to each classifier were logistic regression with l1 penalty C as 2×10^−3^; logistic regression with l2 penalty C as 1×10^−6^; random forest number of estimators as 100, maximum depth as 3, minimum samples per leaf as 50, minimum samples per split as 10, and entropy as the splitting criterion; and multilayer perceptron l2 regularization parameter α as 15, architecture as 1 hidden layer with 5 units, and learning rate as 0.001. Five-fold stratified cross validation on the development set yielded AUC for logistic regression with l1 penalty average 0.830±0.006, logistic regression with l2 penalty 0.796±0.007, random forest 0.828±0.007, and multilayer perceptron 0.841±0.005. In an identical experiment for which an AKI event was considered a positive sample regardless of AKI presence in the most recent stay, we had 4592 features per sample with the same code precision. Five-fold stratified cross validation on the development set with identical settings for the hyper-parameters yielded AUC for logistic regression with l1 penalty average 0.850±0.004, logistic regression with l2 penalty 0.819±0.006, random forest 0.853±0.004, and multilayer perceptron 0.853±0.006. DISCUSSION/SIGNIFICANCE OF IMPACT: Our objective was to investigate the feasibility of using machine learning methods on EHR data to provide a personalized risk assessment for “unexpected” AKI in rehospitalized patients. Preliminary model discrimination was good, suggesting that this approach is feasible. Such a model could aid clinicians to recognize AKI risk in unsuspicious patients. The authors recognize several limitations. Since our data set corresponds to a time-window sample, patients with high frequency of hospital utilization are likely overrepresented. Similarly, our data set contains records from only 1 hospital network. Although we supplement with laboratory-based diagnosis, using diagnosis codes as labels is problematic as numerous reports suggest low sensitivity of codes for AKI. Future work includes calibration analysis, incremental updating (“online learning”), and a representation learning-based (“deep learning”) extension of the model.

